# Development of the Binary and Ternary Atorvastatin Solid Dispersions: *In Vitro* and *In Vivo* Investigations

**DOI:** 10.1155/2021/6644630

**Published:** 2021-09-04

**Authors:** Elahe Faraji, Mojdeh Mohammadi, Mohammad Mehdi Mahboobian

**Affiliations:** ^1^Department of Pharmaceutics, School of Pharmacy, Hamadan University of Medical Sciences, Hamadan, Iran; ^2^Department of Pharmacology & Toxicology, School of Pharmacy, Hamadan University of Medical Sciences, Hamadan, Iran

## Abstract

The object of this study was to prepare binary and ternary solid dispersions of atorvastatin (ATR) by the melting method using PEGs and poloxamer 188 (P188) as the carriers, singly and in combination with each other. Dissolution behavior, solubility studies, X-ray diffractometry, differential scanning calorimetry, and Fourier transform infrared spectroscopy were studied. Furthermore, antihyperlipidemic activities of formulations were compared to each other by serum lipid analyses in hyperlipidemic rats. Based on the results, the highest dissolution efficiency (DE30 = 83%) was obtained by binary systems consisted of ATR and P188. Also, no additional improvement was observed in dissolution properties of ternary solid dispersion formulations. Solubility studies showed enhancement of ATR phase solubility in water and a buffer solution containing P188 or PEG 10000. Furthermore, saturated solubility of ATR in the buffer solution improved more than twofold in the optimized ternary dispersion system. No crystalline changes occurred in PEG-based formulations; meanwhile, partial amorphization happened in the ATR-P188 combination. Finally, the in vivo study in hyperlipidemic rats exhibited a rapid decrease in the lipid profile of all formulations compared to ATR (after 7 days). Moreover, reduction of serum triglycerides and total cholesterol on the 14th day in the ATR group (*p* value < 0.01) was less than solid dispersion or physical mixing preparations (*p* value < 0.001). These findings proved the appropriate influence of using PEG and P188 in solid dispersion systems for the improvement of the therapeutic efficiency of ATR.

## 1. Introduction

Hyperlipidemia or dyslipidemia is a chronic condition that refers to an abnormality in total cholesterol (TC), triglyceride (TG), high-density lipoprotein (HDL), and low-density lipoprotein (LDL) concentration in the blood and usually can be diagnosed by isolated elevation of TG, TC, or both. The relationship between the blood lipid level and the risk of cardiovascular diseases (CVD) such as myocardial infarction and stroke, as the leading cause of death in adults, is well established. Approximately, those with dyslipidemia are at a twofold risk for CVD comparing to those with standard conditions. Many medications were approved to control hyperlipidemia, including statins, bile acid sequestrates, fibric acid derivations, niacin, and ezetimibe [1–3].

Atorvastatin (ATR), the most prescribed statin, is a selective inhibitor of the HMG-CoA reductase and is currently used as calcium salt to treat hyperlipidemia [4]. The negative point of ATR is low oral bioavailability (12%), due to its poor solubility, that results in high dose administration and more adverse effects [5].

Nowadays, the insufficient aqueous solubility of most drugs is an essential challenge in drug delivery. More than 70% of drugs undergoing development and 40% of new chemicals have poor water solubility leading to limited dissolution rate and bioavailability [6, 7]. Most of the drugs with water solubility problem, like ATR, are classified in the Biopharmaceutical Classification System (BCS) as class II, which have good permeability but low solubility [8]. Due to low aqueous solubility, numerous techniques have been developed to overcome this deficiency such as particle size reduction, complexation, micelles, salt formation, prodrug formation, microemulsions, nanoemulsions, nanosuspension formation, solid lipid nanoparticle, and solid dispersion development [9, 10]. Among the mentioned methods, solid dispersion (SD) is one of the most influential and standard methods because of its simplicity in preparation and optimization [11]. SD is explained as the dispersion of an active substance in an inert vehicle. This strategy leads to enhancement of solubility and the dissolution rate of drugs by different mechanisms, including particle size reduction, wettability improvement, and conversion of crystalline to an amorphous form of the drug [12, 13].

Miscellaneous methods are used for SD formulations, including melting, solvent evaporation, lyophilization, extruding technology, electrostatic spinning, the supercritical fluid, and the spray-dried techniques [14]. It is worth mentioning that each process for SD preparation has some limitations. In the solvent evaporation method, the remaining organic solvent in the final sediment can cause toxicity. In addition, API conversion into different polymorphic forms with varying properties of dissolution can hinder reproducibility. These issues also happen for any other solvent-dependent approaches [15]. The spray-dried method requires more equipment. It should be noted that machine configuration may influence different formulations that could affect SD properties [16]. In the melting method, usually, the drug disperses in the molten carrier, or in some cases, both medication and carrier are mixing in the molten state, which is further solidified by cooling even at room temperature or by ice bath [17]. The limitation of the hot-melt method is stability, so it is better to choose carriers with lower melting points. Thus, the melting method was selected for this study due to simplicity in the steps of processing, absence of organic solvent, having a much finer dispersion, and being less time-consuming [14, 18].

For the development of SDs, various carriers have been employed. Among them, polyvinylpyrrolidone, hydroxypropyl methylcellulose, lactose, polyethylene glycol, myrj 52, sorbitol, mannitol, urea, and beta cyclodextrin are the most commonly used [19–27].

Nowadays, nonionic surfactants have been drawing great attention to improve solubility due to their comparatively low toxicity and high solubilizing ability [28]. Poloxamers or Pluronics® are directed from such categories and are formed from polar (polyethylene oxide) and nonpolar (polypropylene oxide) blocks, which lead to being named as a triblock copolymer and generating water solubility, amphiphilic nature, and surface-active properties for this family [29].

Various grades of this type of surfactant, such as poloxamer 188 (P188), are used to promote the dissolution rate of several drugs because of great dispersion, low melting point, good solubilizing capacity, safety, and preventive properties of particle aggregation [11, 30].

Polyethylene glycols (PEG) are long-chain polymers composed of repeated ethylene glycol units and most commonly used in SD formulations as hydrophilic carriers. They have been shown to increase the dissolution profile of a wide range of drugs. PEG is available in different molecular weights, which are suitable for SD because of their solubilizing and wetting properties [31–33].

Some studies have been conducted regarding the improvement of ATR dissolution through SD. For instance, the use of PEG 4000 as a carrier by fusion and PEG 6000 by dropping methods showed a significant increase in solubility and dissolution rate comparing to both the physical mixture and pure ATR [34, 35]. Also, poloxamer 188 was recently used as a carrier in ATR solid dispersion by the conventional solvent evaporation method and revealed satisfactory improvement in both the dissolution profile and bioavailability [16]. Among previous investigations through SD development of ATR, there is a lack of comprehensive study for effectiveness evaluation of binary (polymer or surfactant with ATR) and ternary systems (combination of polymer and surfactant with ATR). Therefore, the present study was aimed at developing ATR binary and ternary SDs by employing different PEG molecular weights (4000 or 10000)and poloxamer 188 as SD carriers in different ratios using the fusion method, and assessment of *in vitro* and *in vivo* performance of selected formulations.

## 2. Materials and Methods

### 2.1. Materials

Atorvastatin calcium (ATR) was obtained from Alborz Bulk Pharmaceutical Co. (Tehran, Iran). PEG 4000 and 10000 were purchased from Samchun Pure Chemical Co., Ltd. (Seoul, Korea) and Exir Co. (Wein, Austria), respectively. Poloxamer 188 was purchased from Johnson Matthey Co. (London, England). Cholesterol extra pure and colic acid were purchased from Merck (Darmstadt, Germany) and Sigma-Aldrich (Germany), respectively. Diagnostic kits for triglyceride, cholesterol, and HDL were purchased from Pars Azmoon Co. (Tehran, Iran). All other materials were of pharmaceutical grades.

### 2.2. Preparation of ATR Solid Dispersions (SDs) with Binary and Ternary Systems

To prepare binary solid dispersion (BSD) of ATR by different PEGs as a polymer (PEG 4000 or PEG 10000), and poloxamer A188 as the surfactant and ternary solid dispersion (TSD) containing the combination of polymer and surfactant, the melting method was used. In the first step, each carrier (PEG 4000, PEG 10000, and poloxamer 188) was heated individually in the water bath (Memmert ONE 10, Germany) to its melting temperature at about 55-60°C for PEG 4000, 62-65°C for PEG 10000, and 56-57°C for poloxamer 188. Then, ATR dispersed in the molten carrier with constant stirring using a mortar and pestle, and finally, the melted drug-carrier mixture was cooled immediately at room temperature. Formulations were kept in a desiccator for 48 h at about 25°C, then ground in the mortar and passed through sieve no. 100 (orifice size: 150 *μ*m) to make identical particle sizes [34]. Drug-polymer BSDs were formulated in various weight ratios of the drug : PEG, involving 1 : 1, 1 : 3, 1 : 5, and 1 : 7 for both PEG 4000 and PEG 10000, whereas drug-surfactant BSDs were formulated in drug : poloxamer 188 weight ratios of 1 : 1, 1 : 3, and 1 : 5.

According to the prior studies, the suitable ratios of BSDs were selected to develop ternary solid dispersion (TSD) of atorvastatin with PEG 10000 and poloxamer 188 as a surfactant in various drug-surfactant-polymer weight ratios of 1 : 1 : 5, 1 : 3 : 5, and 1 : 5: 5. TSDs were produced by adding a surfactant (poloxamer 188) to PEG 10000 in the molten state, and after molting both polymer and surfactant, ATR dispersed in the molten mixture. The rest of the procedure was performed as described.

### 2.3. Preparation of Physical Mixtures

Physical mixtures (PM) of binary and ternary systems were made by uniform blending of ATR and carriers, which were previously pulverized and sieved through mesh no. 100, in a porcelain mortar to get a uniform mixture. They were kept in a desiccator at room temperature for 48 h [19].

### 2.4. *In Vitro* Dissolution Studies

Dissolution studies of the formulations (equivalent to 20 mg of ATR) were performed in a 250 ml phosphate buffer solution (pH = 6.8) at 37 ± 0.5°C for one hour using USP type I (basket) automated dissolution test apparatus. Samples were withdrawn at 5, 10, 15, 20, 30, 40, 50, and 60 minutes and replaced with the same volume of fresh dissolution buffer to maintain sink condition. Samples were centrifuged at 13000 rpm for 10 min, and then, the supernatant was separated and diluted. Samples were analyzed spectrophotometrically at 242 nm in the UV-VIS spectrophotometer. For comparing the dissolution behavior of all formulations, the dissolution efficiency (DE) of each sample after 30 min was calculated using the trapezoidal rule as expressed in the following equation:

(1) $$\begin{array}{c} \text{DE}=\frac{\int_{0}^{t}y\times dt}{y100\times t}\times 100, \end{array}$$

where *y* is the percentage of drug dissolved at time *t*, the numerator shows the area under the dissolution curve till time *t*, and the denominator shows the area until time *t*.

### 2.5. Solubility Studies

#### 2.5.1. Phase Solubility Studies

The solubility of ATR in PEG 10000 and P188 solutions was examined alone and also in association with each other, both in water and PBS. Herein, aqueous and phosphate solutions containing different concentrations (2.5%, 5%, 7.5%, and 10% *w*/*v*) of each carrier (PEG 10000 and P188) and also different concentrations containing the combination of both carriers (P188 : PEG 10000; 2.5% : 10%, 5% : 10%, 7.5% : 10%, and 10% : 10% *w*/*v*) were prepared, and an excess amount of pure ATR was added to 1 ml of each solution. Then, samples kept in a shaker incubator at 37 ± 0.5°C for 48 h. In the end, samples were centrifuged, diluted, and assayed spectrophotometrically at 242 nm as before [36].

#### 2.5.2. Saturated Solubility Studies

Saturated solubility was done for optimum SD formulations and their physical mixtures besides untreated ATR in both water solution and phosphate buffer solution (PBS). Excess amount of untreated ATR, SDs, and PMs was added in the test tube containing 1 ml phosphate buffer and also in the separate test tubes containing 1 ml distilled water, and then, they were kept in a shaker incubator for 48 h at 37 ± 0.5°C. Then, they were centrifuged, diluted, and analyzed for ATR content at 242 nm as mentioned before [37].

### 2.6. Solid State Characterization

#### 2.6.1. Powder X-Ray Diffractometry (PXRD)

The XRD study was employed for pure ATR, surfactant (P188), and polymer (PEG 10000) and obtained BSD by surfactant, BSD by polymer, and TSD containing both polymer and surfactant and also corresponding physical mixtures to determine the crystalline state of each sample using an X-ray diffractometer (Malvern Panalytical BV, Netherland). The X-ray source emits CuK*α* radiation (*k* = 1.5406 Å) filtered through nickel and performed at a voltage of 40 kV and 40 mA current. The anode material was Cu, and samples were scanned over a 2Ө range of 2-70° at a step size of 0.026° and step time of 37.995 s.

#### 2.6.2. Differential Scanning Calorimetry (DSC)

Thermal characteristics of pure ATR, poloxamer 188, PEG 10000, selected SDs, and their relative physical mixtures were recorded using DSC 823 (Mettler-Toledo International Inc., Switzerland). About 5-7 mg of each sample was weighed and sealed in a standard aluminum pan. A sealed empty aluminum pan was used as a reference. Both pans were heated over a temperature range of 20 to 200°C at a heating rate of 10°C/min. Air-gas purge was used to maintain an inert atmosphere in sample cells [38].

#### 2.6.3. Fourier Transform Infrared (FTIR) Spectroscopy

Infrared spectroscopy was carried out for optimized formulations obtained from BSD, TSD, corresponding physical mixtures, and SD components using an FTIR spectrometer (Bruker Alpha, Tensor 27, Germany) to investigate any probable chemical interaction between carries and ATR. Samples were analyzed using the KBr disk method. For this, a soupcon of each sample was triturated adequately with potassium bromide to obtain fine homogenous powder and then compressed into a thin and transparent disk. Each sample was scanned through a wave number range of 4000-400 cm^−1^ at a resolution of 2.0 cm^−1^ [39].

### 2.7. Animal and Experimental Design

The animal study was performed in accordance with the Guideline of the Institutional Animal Ethics Committee of the Hamadan University of Medical Science, Hamadan, Iran (IR.UMSHA.REC.1397.1018; 2019.03.16). The hypolipidemic activity of optimum formulations of BSD with P188 (1 : 1, drug : P188 ratio) and BSD with PEG 10000 (1 : 5, drug : PEG ratio) and TSD by both carriers (1 : 1 : 5, drug : P188 : PEG ratio) was determined in comparison with their physical mixtures and pure ATR in healthy albino rats. Male Wistar rats weighing 170-200 g were housed in cages on a 12 h light/dark cycle at a controlled temperature of 25°C with a relative humidity of 50 ± 10%, free access to tap water, and a pelleted diet. All animals were adapted to laboratory conditions for at least one week. In order to induce hyperlipidemia, each group received daily a high-fat diet (HFD) regimen prepared by mixing 1 mg/kg cholesterol and 0.05 mg/kg cholic acid in 7.5 mg/kg coconut oil. The high-fat diet regimen was administrated orally using gavage feeding needles. The control group was fed only by HFD regimen; other groups were treated with SD formulations and their corresponding PMs equivalent to 5 mg/kg/day ATR orally. The treatment was given for 14 days [38, 40–42]. Blood samples were collected using light ether anesthesia by eye puncture initially (before starting the experiment) and then after 7 and 14 days. The serum was separated by centrifugation of blood at 4000 rpm for 10 min and analyzed for concentration of triglycerides (TG), total cholesterol (TC), and high-density lipoprotein-cholesterol (HDLc) using an in vitro diagnostic kit (Pars Azmoon Co., Iran) [40, 42, 43].

The animals were divided into eight groups of six as follows:

- *Group 1*: hyperlipidemic control, which received just lipid mixture
- *Group 2*: hyperlipidemia treated with SD of ATR : P188 (1 : 1 ratio) 10 mg/kg/day
- *Group 3*: hyperlipidemia treated with PM of ATR : P188 (1 : 1 ratio) 10 mg/kg/day
- *Group 4*: hyperlipidemia treated with SD of ATR : PEG 10000(1 : 5 ratio) 30 mg/kg/day
- *Group 5*: hyperlipidemia treated with PM of ATR : PEG 10000 (1 : 5 ratio) 30 mg/kg/day
- *Group 6*: hyperlipidemia treated with SD of ATR : P188 : PEG 10000 (1 : 1 : 5 ratio) 35 mg/kg/day
- *Group 7*: hyperlipidemia treated with PM of ATR : P188 : PEG 10000 (1 : 1 : 5 ratio) 35 mg/kg/day
- *Group 8*: hyperlipidemia treated with pristine ATR 5 mg/kg/day

### 2.8. Statistical Analysis

In this study, all the experiments were repeated in triplicate and reported as the mean value ± standard deviation (SD). Statistical data were analyzed using one-way analysis of variance (ANOVA) followed by a post hoc test (Tukey's test) using GraphPad Prism 7, and the *p* value less than 0.05 was considered to be statistically significant.

## 3. Results and Discussion

### 3.1. In Vitro Dissolution Studies

**Figure 1:**
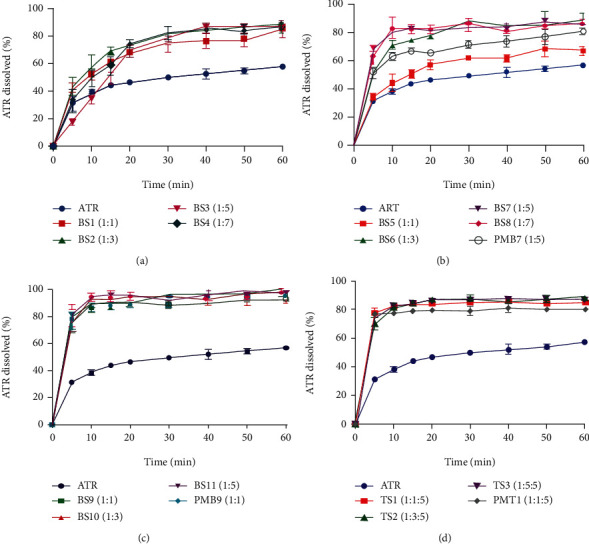
Dissolution profiles of untreated drug (ATR), binary solid dispersion (BS), ternary solid dispersion (TS), and relevant physical mixture (PM) with different drug : carrier ratios: (a) ATR : PEG 4000, (b) ATR : PEG 10000, (c) ATR : poloxamer 188, and (d) ATR : poloxamer 188 : PEG 10000 (*n* = 3).

To develop new formulations amid various ones and their comparison, the dissolution study could be used as the first distinct analysis to choose the best formulations [44]. The dissolution profiles obtained for BSD with PEG 4000, PEG 10000, and poloxamer 188 in various drug : polymer ratios are shown in Figure 1. All formulations, either SDs or PMs, showed, obviously, enhancement in the drug dissolution rate. As shown in Table 1, there was a significant difference between DE_30_ obtained from BSD formulations with PEG 4000 (BS1-BS4) and BSD formulations with PEG 10000 (BS5-BS8) compared to the intact drug. Maximum DE_30_ was observed in 1 : 3 and 1 : 7 SD ratios for PEG 4000 and 10000, which shows about 21% and 34% increases compared to the drug powder, respectively. It seems that the application of the hydrophilic polymer as PEG could potentially increase the dissolution characteristic of ATR. High polarity of PEG can decrease the contact angle of drug particles with the dissolution medium, which results in hydrophilicity elevation and, hence, solubility acceleration. Nonetheless, applying PEG 10000 was significantly (*p* < 0.001) more effective than PEG 4000 (with 30% higher DE_30_) on drug dissolution, which may be due to the larger hydration capacity of PEG 10000. Thus, improvement of the ATR dissolution rate happened because of decreasing contact angle, increasing hydrophobic interaction of the polymer and hydrophobic drug, and also the reduction of the polarity of the solvent system [33]. This trend has been reported in previous works using PEG 6000 and 10000 for irbesartan and PEG 6000 and 12000 for simvastatin [45, 46]. In BSD with PEG 10000, formulations named BS7 and BS8 (1 : 5 and 1 : 7, drug : PEG ratio, respectively) were shown to have a significant higher DE_30_ value than formulations with a lower ratio of PEG. The dissolution rate of ATR increased as part of PEG grew, while there was no significant difference between the 1 : 5 and 1 : 7 ratios (BS6 and BS7, respectively, *p* value > 0.05). This event might be related to the higher viscosity of the PEG layer around the drug and the reduction of the diffusion coefficient that can hinder drug release from the solid state [47]. Besides, the physical mixture containing a 1 : 5 drug : polymer ratio was prepared and subjected to the dissolution test. Results showed that the dissolution rate of ATR from PMB7 was significantly higher than that of the intact drug but lower than that of the same ratio of SD (BS7), which could be seen within 10 min (Figure 1). It shows that PEG 10000 potentially can affect dissolution profiles due to its intrinsic properties, as mentioned before. Furthermore, inhibition of drug particle aggregation by improving drug wettability is considered as another mechanism for dissolution enhancement in PM formulations [27]. Despite that, the SD process had an extra effect on dissolution enhancement, maybe due to drug recrystallization during SD preparation and molecularly dispersion of the drug in the carrier (PEG 10000), which can cause particle size reduction and surface area elevation that results in better wettability of drug particles [12].

The results from BSD by poloxamer 188 as a nonionic surfactant, exhibited about 40% (twofold) increase in DE_30_ in all formulations by either dispersion in the poloxamer or physical mixing by the poloxamer, comparing with the intact drug. BSD with poloxamer 188 was even better than BSD with PEG 10000 with 10% more efficiency. The benefit of surfactant systems is that solubilization can happen through interfacial processes such as floatation, wetting, and surface-active properties, which reduce interfacial tension, cause better drug wettability, and prevent particle aggregation [14, 30]. There was no significant difference between BS9, BS10, and BS11 (1 : 1, 1 : 3, and 1 : 5, ATR : poloxamer ratio, respectively), which represents that further increases in the poloxamer component did not have an additional enhancement in ATR dissolution. This could be attributed to forming a viscose matrix due to high quantities of the P188 [14]. The Stokes–Einstein equation expresses that viscosity increase of the dissolution medium decreases the diffusion coefficient and results in lower drug diffusivity and dissolution rate [48].

To investigate the effect of TSD (including the combination of both carriers) on the dissolution rate, the PEG 10000 proportion was kept constant (1 : 5 ATR : PEG ratio), and the proportion of the poloxamer increased in the TSD mixture. As shown in Table 1, results from TSDs (TS1, TS2, and TS3) did not show any extra improvement in the dissolution rate comparing with ATR-PEG 10000 BSD (BSD7) and BSDs with poloxamer. Furthermore, similar to ATR-P188 BSDs, increase in surfactant concentration did not offer further efficiency on the dissolution profile. Using a combination of surfactant and PEG in TSDs increases viscosity more than BSDs, which could hinder burst drug release because PEG and poloxamer need more time to dissolve and act as a control release structure by swelling [49]. Another mechanism in systems containing P188 could be related to the transition from solution to gel state that happens around 37°C, which increases viscosity, causes swelling, and makes a sustained release pattern [29]. In both ATR-poloxamer and ATR-poloxamer-PEG SDs, there were no significant differences compared to their physical mixtures. This also might be related to the high viscous hydrophilic layer of systems containing P188 in the microenvironment surrounding drug particles. Hence, these formulations need more time to show their increasing solubility capacity. Furthermore, during the fusion method, deep incorporation of the drug into the viscose polymeric network of P188 can occur, while in the physical mixture, they were just mixed physically, so SD formulations did not show a significant increase in DE_30_ value compared to PMs, statistically [27].

### 3.2. Solubility Studies

#### 3.2.1. Phase Solubility

ATR solubility in water and phosphate solution containing different carrier concentrations is represented in Table 2. Based on the results, ATR solubility was improved in hydrophilic carriers, but there were differences in the maximum amount of solubilized ATR. In solutions containing 10% PEG 10000, drug solubility indicated approximately 2- and 3-fold increases in the water and buffer solutions, respectively. Results prove that PEG concentration had a linear effect on ATR solubility. This is concurrent with the results from the dissolution rate study of ATR-PEG formulations and the mechanism mentioned before.

The same result was achieved for ATR solubility at different concentrations of poloxamer 188 solutions. By increasing poloxamer 188 concentrations, the phase solubility of ATR increased. It should be noted that ATR had superior solubility in poloxamer 188 solutions comparing with PEG solutions. Similar results were obtained by Mura et al. in binary and ternary SDs of naproxen [50]. This revealed that poloxamer 188 had a more significant solubilizing effect due to surfactant properties. Moreover, self-assembling of poloxamer 188 to micelles can cause more solubilization of the drug through the micellar solubilizing ability, while for the PEG solution, only wettability of the drug improved [51].

Results from solutions containing a combination of PEG 10000 and poloxamer 188 were noteworthy. As mentioned before, in solutions containing both carries, the concentration of PEG was kept constant at 10% *W*/*V* and the portion of poloxamer was varied (Table 2). In phosphate and water solutions containing both surfactant and PEG, by increasing the poloxamer concentration to 10% *W*/*V*, the ATR phase solubility decreased. Adding surfactant (P188) to the PEG solution could hinder the increased ATR solubility caused by PEG. This also accurately confirms the effect of the high viscosity of medium generated by the high concentration of PEG plus poloxamer. Thus, an extremely high concentration of carries resterics drug solubilization. The results exactly confirm the data obtained from the dissolution study.

#### 3.2.2. Saturated Solubility

Saturated solubility was also performed in water and phosphate buffer.  Table 3 shows the results of saturation solubility for selected formulations. Our findings indicated that ATR had better saturated solubility in the phosphate buffer than water. In the binary system by PEG, solid dispersion formulation (BS7) significantly shows higher saturated solubility than the corresponding physical mixture (PMB7). In contrast, in a binary system with poloxamer, there isn't any superiority influence of the SD system (BS9) on saturated solubility versus its relevant physical mixture (PMB9). These findings prove the events mentioned for the dissolution rate of the ATR-PEG and ATR-P188 combinations. In ternary formulations, both SD (TS1) and the physical mixture (PMT1) showed a significant increase in saturated solubility, which was even better than both BSDs that seem to be related to a high solubilizing capacity due to the existence of both the polymer and surfactant.

### 3.3. Solid State Characterization

#### 3.3.1. Powder X-Ray Diffraction (PXRD)

**Figure 2:**
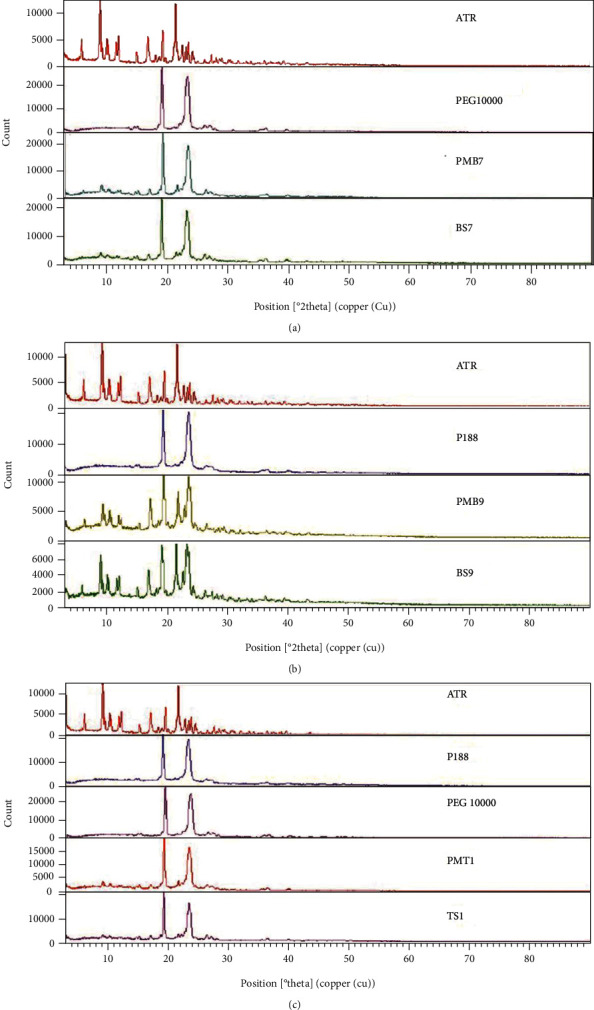
PXRD spectra of (a) PEG binary systems, (b) P188 binary systems, and (c) ternary systems. ATR: untreated atorvastatin; PEG 10000: polyethylene glycol 10000; P188: poloxamer 188; PMB7: physical mixture (ATR : PEG 10000, 1 : 5); BS7: solid dispersion (ATR : PEG 10000,1 : 5); PMB9: physical mixture (ATR : P188, 1 : 1); BS9: solid dispersion (ATR : P188, 1 : 1); PMT1: physical mixture (ATR : P188 : PEG 10000, 1 : 1 : 5); TS1: solid dispersion (ATR : P188 : PEG 10000, 1 : 1 : 5).

The powder X-ray diffraction studies are helpful for further characterizing the sample crystalline state and discovering any crystalline modification during processes. The PXRD diffractograms of pure ATR, PEG 10000, poloxamer 188, BSDs, TSD, and their physical mixtures are depicted in Figure 2. ATR peaks were detected at the 2Ө scattered angle of 9.1, 10.2, 12.1, 17.01, 18.24, 19.4, 21.5, 22.6, 23.6, and 27.4, and also several sharp and intense peaks were detected at the 2Ө scattered angle of 19.2 and 23.33 for PEG 10000, which are related to its crystalline form [16, 45]. The BS7 and PMB7 diffractograms indicated the XRD crystalline peaks for both PEG 10000 and pure ATR at the same 2Ө values, which showed that the drug remained crystalline in BSD and relative PM, and no amorphization occurred during processes. Although the intensity of the ATR peak decreased significantly in both PMB7 and BS7 formulations, the intensity of PEG remained as strong as before, which could be due to a lower percentage of ATR in the formulation compared to the carrier (1 : 5, ATR : PEG ratio). This finding showed that increase in the dissolution rate of ATR from BSD containing PEG is not related to the crystalline changes and may be due to the other factors mentioned in Section 3.1 [46].

The bulk P188 showed a crystalline pattern with intense characteristic peaks at a 2Ө diffraction angle of 19.2 and 23.5. The percentage of crystallinity decreased from 18% in pure ATR to 15% in both BS9 and PMB9 formulations (1 : 1, ATR : P188 ratio), although the XRD peaks of untreated ATR and P188 could be detected in both SD and the PM samples. This finding revealed that dissolution and solubility increase in the ATR-P188 combination also could be attributed to slight amorphization in both SD and PM. So the presence of poloxamer could affect dissolution and solubility by amorphization in addition to previously mentioned mechanisms.

Figure 2(c) illustrates the PXRD pattern for TS1 and PMT1 (1 : 1 : 5, ATR : P188 : PEG ratio) comparing to their components. The PM and SD samples show peaks of the PEG 10000 and P188, in addition to some low-intensity diffraction peaks relating to the bulk drug, which displays no amorphization in the ternary formulation containing both polymer and surfactant.

The XRD patterns of all PEG-containing formulations showed that not only no amorphization occurred, but also, crystallinity was increased about 3% in BS7 and 4% in PMB7 and PMB9 that could be related to more crystallinity of PEG than the pure drug.

#### 3.3.2. Differential Scanning Calorimetry (DSC)

**Figure 3:**
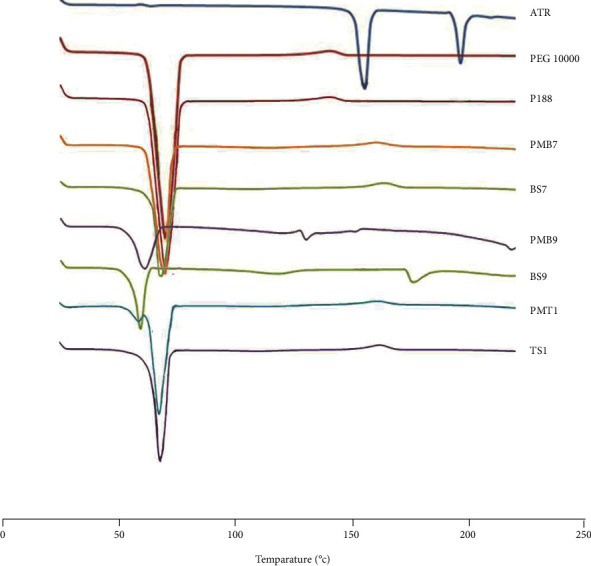
DSC thermograms of ATR: untreated atorvastatin; PEG 10000: polyethylene glycol 10000; P188: poloxamer 188; PMB7: physical mixture (ATR : PEG 10000, 1 : 5); BS7: solid dispersion (ATR : PEG 10000, 1 : 5); PMB9: physical mixture (ATR : P188, 1 : 1); BS9: solid dispersion (ATR : P188, 1 : 1); PMT1: physical mixture (ATR : P188 : PEG 10000, 1 : 1 : 5); TS1: solid dispersion (ATR : P188 : PEG 10000, 1 : 1 : 5).

DSC examination was applied to study the crystalline state of selected SD formulations and their PMs and any interaction between components in either SDs or PMs [52].  Figure 3 represents the DSC thermograms of prepared SDs and their corresponding PMs in comparison with their components. The DSC curve of P188 and PEG 10000 exhibited a sharp endothermic peak at 55.36°C and 64°C, respectively, related to the melting point of each component [16, 45]. The DSC curve of ATR displayed two endothermic peaks: the first endothermic peak at 150.39°C is related to the melting point of ATR and the second peak at 191.73°C may be due to the degradation product of ATR [53]. Based on the thermograms, the drug endothermic peak completely disappeared in both BS7 and PMB7 formulations, and only a single peak corresponding to PEG 10000 was recorded without any changes in melting temperature, which confirms that the disappearance of the ATR endothermic peak is not related to amorphization (as XRD proved), and it could be related to the dissolution of ATR in PEG when it melted during DSC examination for both BSD7 and PMB7 formulations [46].

The thermal profile of TS1 (1 : 1 : 5, ATR : P188 : PEG ratio) only showed a single PEG 10000 endothermic peak with a complete disappearance of the P188 and ATR peaks. In the PMT1 thermogram, a small and short peak has emerged neighbouring the PEG 10000 endothermic peak, which could be related to P188 in terms of its melting temperature and cause the main peak (PEG 10000) to be shortened diagrammatically. It seems that in the TS1 thermogram, the P188-related peak was incorporated to the PEG 10000 peak because of high dispersion during the melting and mixing processes; on the contrary, in the PMT1 formulation, P188 has not dispersed as well as TS so that a short peak was recorded because of the lower percentage of P188 compared to PEG.

The thermal profile of BS9 (1 : 1, ATR : P188 ratio) and PMB9 formulations recorded a sharp peak belonging to the P188 melting temperature with a slight shift (decreasing melting point), which proves minor amorphization in both samples, which precisely corresponded to the result from the XRD analysis [54].

#### 3.3.3. FTIR Spectroscopy

**Figure 4:**
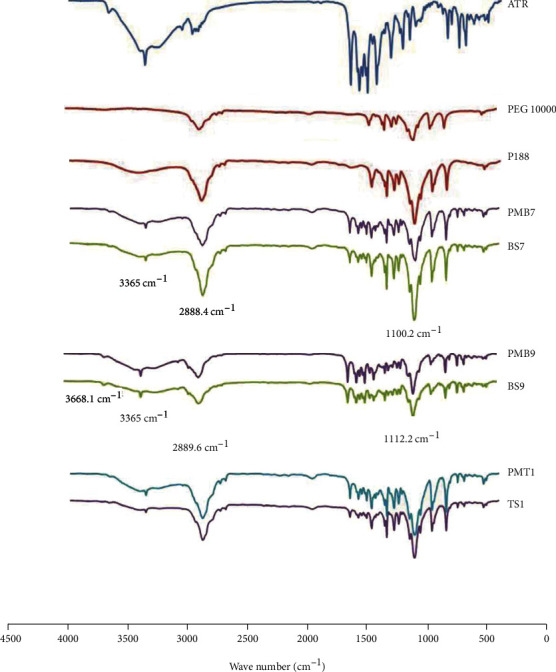
FTIR spectra of ATR: untreated atorvastatin; PEG 10000: polyethylene glycol 10000; P188: poloxamer 188; PMB7: physical mixture (ATR : PEG 10000, 1 : 5); BS7: solid dispersion (ATR : PEG 10000, 1 : 5); PMB9: physical mixture (ATR : P188, 1 : 1); BS9: solid dispersion (ATR : P188, 1 : 1); PMT1: physical mixture (ATR : P188 : PEG 10000, 1 : 1 : 5); TS1: solid dispersion (ATR : P188 : PEG 10000, 1 : 1 : 5).

Concerning any interaction between carriers with each other or drug during processes that affect chemical bonds, IR spectroscopy is a useful tool for finding these modifications.  Figure 4 exhibits the FTIR spectra of pure ATR, PEG 10000, poloxamer 188, SDs, and corresponding PMs. As shown, the spectrum of ATR showed sharp characteristic peaks at 3668.1 cm^−1^ (related to the free O-H stretching vibration), 3365 cm^−1^ (related to the N-H stretching bond), 3270.7 cm^−1^ and 3056 cm^−1^ (asymmetric and symmetric O-H stretching, respectively), 2971.3 cm^−1^ (related to the C-H stretching), 1650.7 cm^−1^ and 1579.3 cm^−1^ (representing asymmetric and symmetric C=O stretching, respectively), 1510.2 cm^−1^ and 1435.9 cm^−1^ (representing C-C aromatic stretching), 1316.9 cm^−1^ (showing deformation of CH_3_/CH_2_ bonds), 1217.1 cm^−1^ (related to C-N stretching bonds), and 1159 cm^−1^ (related to C-F stretching bond). The spectrum of PEG 10000 shows the prominent characteristic peaks at 2888.4 cm^−1^ (related to C-H stretching vibration) and 1100.2 cm^−1^ (related to C-O stretching bonds). The typical peaks of P188, which are related to the main function groups recorded at 2889.6 cm^−1^, are related to C-H stretching bonds and 1112.2 cm^−1^ that represent C-O groups [16, 55].

All characteristic peaks of ATR and PEG 10000 appeared in both BS7 and PMB7 formulations with no significant changes in the wavenumber comparing to their particular spectrum, and also, the absence of any additional peak in both BS7 and PMB7 formulations affirms no chemical interaction between PEG and ATR. The spectrum of the binary formulation containing P188 (BS9 and PMB9) also showed the prominent peaks of their components without any additional peak which indicated no interaction between ATR and P188. The same result happened for ternary formulations that revealed no chemical interaction between the polymer and surfactant (PEG 10000 and P188) [55].

### 3.4. Antihyperlipidemic Activity in Rats

**Figure 5:**
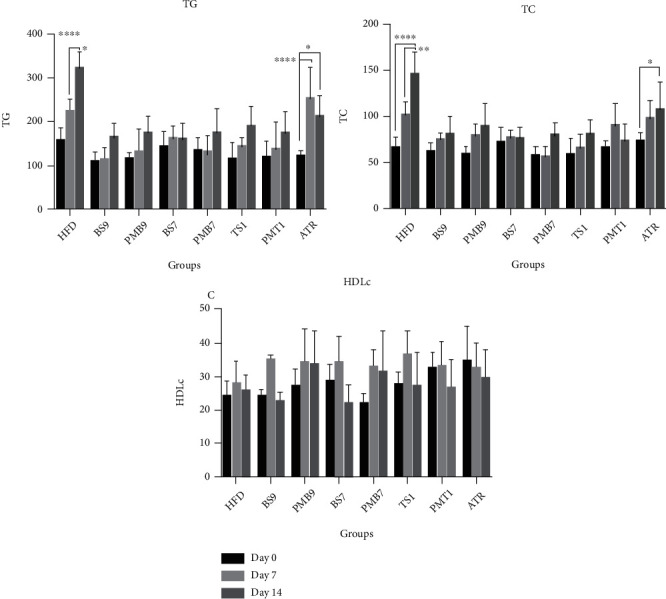
Antihyperlipidemic activity in rats. TG: triglyceride; TC: cholesterol; HDLc: high-density lipoprotein cholesterol; HFD: high-fat diet; ATR: untreated atorvastatin; PEG 10000: polyethylene glycol 10000; P188: poloxamer 188; PMB7: physical mixture (ATR : PEG 10000, 1 : 5); BSD7: solid dispersion (ATR : PEG 10000, 1 : 5); PMB9: physical mixture (ATR : P188, 1 : 1); BSD9: solid dispersion (ATR : p188, 1 : 1); PMT1: physical mixture (ATR : P188 : PEG 10000, 1 : 1 : 5); TSD7: solid dispersion (ATR : P188 : PEG 10000, 1 : 1 : 5); BS7: solid dispersion (ATR : PEG10000, 1 : 5); BS9: solid dispersion (ATR : P188, 1 : 1); TS1: solid dispersion (ATR : P188 : PEG10000, 1 : 1 : 5). (Each point represents the mean ± SEM level, ^∗^*p* < 0.05, ^∗∗^*p* < 0.01, ^∗∗∗^*p* < 0.001, and ^∗∗∗∗^*p* < 0.0001, *N* = 6 in each group).

The antihyperlipidemic activity of pure ATR, ternary SD, binary SDs, and their corresponding physical mixtures was assayed by a lipid-lowering study in a high-fat diet-induced hyperlipidemia rat model. As shown in Figure 5, serum TG and TC levels elevated remarkably in the HFD group with a continuous rise in the 7^th^ and 14^th^ days by administrating a high-fat regimen. This finding revealed that the high-fat diet protocol was completely effective in inducing hyperlipidemia. However, there was no significant difference in the serum HDL level of all groups between the beginning of the experiment, the 7^th^ day, and the 14^th^ day, as reported the same by the other researchers [38], whereas in rats treated with formulations (SD or PM), significant effects (*p* value < 0.001) on serum TG and TC after 14 days were observed compared to the HFD group. Also, in pure ATR-treated rats, TG and TC decreased significantly (*p* value < 0.01) after 14 days, but the reduction was less than SD or PM samples. These findings could be related to the rapid dissolving of ATR, leading to quick absorption of all prepared samples compared to the intact drug. In pure ATR-treated rats, serum TG increased on the 7^th^ day significantly more than groups treated with any other formulations (either SDs or PMs) containing a polymer or surfactant slightly regulated in the 14^th^ day, which proves that the low solubility of pure ATR takes a longer time to reach the intended remedial goal, and at this dose of ATR (5 mg/kg/day), more prolonged treatment may be necessary to diminish serum TG levels relatively similar to those formulations containing the polymer or surfactant [43]. The elevation of TC in pure ATR-treated rats was not significant on the 7^th^ day but significant on the 14^th^ day. These findings proved that all prepared formulations were more effective in regulating TC and TG than the intact drug. It should be noted that in all treated formulation groups (either SDs or PMs), there was no significant elevation after 14 days in both TG and TC. Moreover, there was no significant difference between SD formulations and their corresponding PMs in antihyperlipidemic activity, indicating good accordance with *in vitro* studies regarding the similar dissolution behavior of these formulations. Finally, it can be concluded that this combination of hydrophilic carriers, either surfactant or polymer or both with ATR, as an HMG-CoA reductase inhibitor, could affect better on TG and TC regulation.

## 4. Conclusion

The present study demonstrated that solid dispersion of ATR-PEG 10000, ATR-P188, and ATR-P188-PEG 10000 could be effectively prepared by the melting method, which could enhance the dissolution rate and solubility. Among these, ATR-surfactant solid dispersion and its physical mixture had a superior effect in comparison with ATR-polymer due to the more solubilizing capacity of the employed surfactant. Also no additional improvement was observed in the dissolution rate of surfactant-polymer combination, as a consequence of the viscose layer formation which hinder drug release from the solid state. DSC and PXRD analyses revealed no changes in crystallinity of formulations containing PEG, while in ATR-P188, slight amorphization occurred. Additionally, FTIR analysis proved that there were not any chemical interactions between components. Antihyperlipidemic activity of formulations showed a better antihyperlipidemic effect in all formulations containing hydrophilic carriers as solubilizing agents compared to the intact drug. Thus, the correlation between *in vitro* and *in vivo* studies is evident. Finally, it can be concluded that solid dispersion or even physical mixture of the ATR with P188 is sufficient in improving its dissolution characteristic, which leads to better *in vivo* efficiency and reduces the required drug dose and consequently diminishes side effects.

## Figures and Tables

**Table 1:** Composition of samples and the dissolution efficiencies (DE_30_).

Formulation		Component ratio			DE (%) (*n* = 3)
	ATR	PEG 4000	PEG 10000	Poloxamer 188	
ATR	1	—	—	—	38.83 ± 0.58
BS1	1	1	—	—	54.73 ± 1.75
BS2	1	3	—	—	59.93 ± 1.85
BS3	1	5	—	—	47.13 ± 2.09
BS4	1	7	—	—	55.38 ± 3.70
BS5	1	—	1	—	46.22 ± 0.71
BS6	1	—	3	—	67.05 ± 1.23
BS7	1	—	5	—	72.89 ± 1.23
BS8	1	—	7	—	73.23 ± 0.75
PMB7	1	—	5	—	58.70 ± 1.18
BS9	1	—	—	1	80.64 ± 4.01
BS10	1	—	—	3	83.12 ± 3.43
BS11	1	—	—	5	82.81 ± 3.95
PMB9	1	—	—	1	79.32 ± 1.90
TS1	1	—	5	1	75.40 ± 1.10
TS2	1	—	5	3	75.48 ± 0.89
TS3	1	—	5	5	76.15 ± 0.31
PMT1	1	—	5	1	71.80 ± 1.07

B: binary; T: ternary; S: solid dispersion; PM: physical mixture.

**(a):** 

ATR phase solubility in the presence of increasing concentrations of carriers in water (*μ*g/ml) (*n* = 3)			
Concentration (%) (*W*/*V*)	PEG 10000	Poloxamer 188	10% PEG 10000 plus increasing concentrations of poloxamer 188
0%	189.47 ± 22.97	189.47 ± 22.97	378.32 ± 11.68
2.5%	227.76 ± 13.61	273.15 ± 14.14	364.19 ± 29.24
5%	290.09 ± 17.14	379.05 ± 16.76	342.00 ± 32.37
7.5%	347.36 ± 7.23	494.79 ± 28.47	362.26 ± 10.53
10%	378.32 ± 11.68	544.12 ± 31.96	277.52 ± 6.70

**(b):** 

ATR phase solubility in the presence of increasing concentrations of carriers in pH = 6.8 PBS (*μ*g/ml) (*n* = 3)			
Concentration (%) (*W*/*V*)	PEG 10000	Poloxamer 188	10% PEG 10000 plus increasing concentrations of poloxamer 188
0% 2.5%	294.35 ± 27.55	294.35 ± 27.55	940.94 ± 29.45
	471.11 ± 15.31	699.49 ± 57.37	852.75 ± 22.38
5%	659.78 ± 18.29	929.40 ± 5.32	895.19 ± 64.15
7.5%	781.09 ± 53.71	1044.70 ± 92.03	791.62 ± 22.46
10%	940.95 ± 29.45	1083.35 ± 146.55	569.62 ± 56.49

**Table 3:** Saturated solubility of ATR, selected BSDs and TSD, and corresponding PMs.

Sample	ATR saturation solubility (*μ*g/ml) (*n* = 3) in water	ATR saturation solubility (*μ*g/ml) (*n* = 3) in PBS (pH = 6.8)
ATR	189.47 ± 22.97	294.35 ± 27.55
BS7	258.36 ± 25.76	514.32 ± 37.19
PMB7	222.11 ± 10.87	399.23 ± 15.86
BS9	228.23 ± 7.96	437.09 ± 55.01
PMB9	227.32 ± 23.41	490.60 ± 19.04
TS1	272.75 ± 26.48	673.95 ± 67.52
PMT1	269.00 ± 8.10	630.59 ± 11.37

## Data Availability

Data are available on request.
